# AC-ASPECTS, ACh-ASPECTS, and H-ASPECTS: new imaging scales to assess territorial and total cerebral hemispheric ischemic injury

**DOI:** 10.3389/fneur.2024.1397120

**Published:** 2024-07-03

**Authors:** Maria Paz Rodriguez, Shayandokht Taleb, Jenny Ji-hyun Lee, David S. Liebeskind, Jeffrey L. Saver

**Affiliations:** ^1^Department of Neurology, Hospital Maciel, Montevideo, Uruguay; ^2^Department of Neurology, Kaiser Permanente Los Angeles Medical Center, Los Angeles, CA, United States; ^3^Comprehensive Stroke Center and Department of Neurology, David Geffen School of Medicine at UCLA, Los Angeles, CA, United States

**Keywords:** early ischemic changes, early change detection, acute stroke diagnosis, Alberta stroke program early CT score (ASPECTS), hemispheric stroke, anterior circulation acute ischemic stroke, ischemic stroke (IS)

## Abstract

**Background:**

The extent of ischemic injury in acute stroke is assessed in clinical practice using the Acute Stroke Prognosis Early CT Score (ASPECTS) rating system. However, current ASPECTS semi-quantitative topographic scales assess only the middle cerebral artery (MCA) (original ASPECTS) and posterior cerebral (PC-ASPECTS) territories. For treatment decision-making in patients with anterior cerebral artery (ACA) occlusions and internal carotid artery (ICA) occlusions with large ischemic cores, measures of all hemispheric regions are desirable.

**Methods:**

In this cohort study, anatomic rating systems were developed for the anterior cerebral (AC-ASPECTS, 3 points) and anterior choroidal artery (ACh-ASPECTS, 1 point) territories. In addition, a total supratentorial hemisphere (H-ASPECTS, 16 points) score was calculated as the sum of the MCA ASPECTS (10 regions), supratentorial PC-ASPECTS (2 regions), AC-ASPECTS (3 regions), and ACh-ASPECTS (1 region). Three raters applied these scales to initial and 24 h CT and MR images in consecutive patients with ischemic stroke (IS) due to ICA, M1-MCA, and ACA occlusions.

**Results:**

Imaging ratings were obtained for 96 scans in 50 consecutive patients with age 74.8 (±14.0), 60% female, NIHSS 15.5 (9.25–20), and occlusion locations ICA 34%; M1-MCA 58%; and ACA 8%. Treatments included endovascular thrombectomy +/− thrombolysis in 72%, thrombolysis alone in 8%, and hemicraniectomy in 4%. Among experienced clinicians, inter-rater reliability for AC-, ACh-, and H-ASPECTS scores was substantial (kappa values 0.61–0.80). AC-ASPECTS abnormality was present in 14% of patients, and ACh-ASPECTS abnormality in 2%. Among patients with ACA and ICA occlusions, H-ASPECTS scores compared with original ASPECTS scores were more strongly associated with disability level at discharge, ambulatory status at discharge, discharge destination, and combined inpatient mortality and hospice discharge.

**Conclusion:**

AC-ASPECTS, ACh-ASPECTS, and H-ASPECTS expand the scope of acute IS imaging scores and increase correlation with functional outcomes. This additional information may enhance prognostication and decision-making, including endovascular thrombectomy and hemicraniectomy.

## Introduction

The medical and surgical management of patients with anterior circulation cerebral infarcts and large hemispheric infarcts has advanced dramatically over the past decade. For patients with isolated anterior cerebral artery (ACA) occlusions, whose lesions were previously inaccessible, a new generation of retriever and aspiration thrombectomy devices are now being tested in randomized trials. For patients with internal carotid artery (ICA) occlusion and large ischemic cores, previously only treatable with hemicraniectomy, multiple recent trials have demonstrated the benefit of endovascular thrombectomy, with final outcome disability levels limited by the extent of pretreatment injury (1). In addition, preliminary trials and studies have shown promise for multiple additional therapies, including intravenous glyburide, strokectomy (compared with hemicraniectomy), selective cerebral hypothermia, and hyperosmolar therapy (2–4). A barrier to the further development of therapeutics for patients with ACA occlusion and patients with ICA occlusion and large ischemic core is that the only available pragmatic assessment of initial and early ischemic injury extent is the Alberta Stroke Program Early Computed Tomography score (ASPECTS), a clinician-performed, semi-quantitative, 10-point topographic imaging assessment (5–11).

The ASPECTS score has been a foundation for acute stroke therapeutics for the past quarter century since its development in 2000 (11). However, although it was developed to assess early ischemic changes in patients with “acute ischemic stroke of the anterior circulation” (refs 6–11), the ASPECTS scale is constrained in its interrogation of the degree of injury to only a subset of relevant fields - the middle cerebral artery (MCA) territory. It provides no useful information regarding the extent of tissue injury in patients with isolated ACA occlusion. It also provides less than optimal information in patients with ICA occlusion and large ischemic cores. In these patients, the extent of injury beyond the MCA territory, including in ACA, anterior choroidal artery (ACh), and supratentorial posterior cerebral artery fields, contributes independently to worse prognosis and the need for early intervention (12–14). Accordingly, clinical management is guided by volumetric imaging of ischemic injury, incorporating all supratentorial arteries, when available (15–17). However, when true volumetric imaging is not available, the prognostic and decision-making accuracy of pragmatic semi-quantitative topographic scan evaluation potentially may be increased by including ratings of injury in the ACA, AChA, and supratentorial PCA fields.

The objective of this study was to develop and preliminarily validate semi-quantitative topographic assessments of the ACA (AC-ASPECTS) and AChA (ACh-ASPECTS) territories and a total hemispheric score (H-ASPECTS) incorporating MCA, ACA, AChA, and supratentorial PCA ASPECTS ratings.

## Methods

The regions that comprise the new scales were selected based on neuroanatomic and imaging atlases that delineated the cerebral arterial territories (18–21).

### Nomenclature

Two salient ASPECTS scores already are in wide use: the original ASPECTS, assessing the MCA territory, and the PC-ASPECTS, assessing the PCA territory. In the expanded system advanced in the current study, the original ASPECTS score is given the more specific designation of the middle cerebral – ASPECTS score (MC-ASPECTS). The three new, complementary ASPECTS scores developed for the current study are: the Anterior Cerebral Acute Stroke Early Cerebral Topographic Score (AC-ASPECTS); the Anterior Choroidal Acute Stroke Early Cerebral Topographic Score (ACh-ASPECTS); and the Hemispheric Acute Stroke Early Cerebral Topographic Score (H-ASPECTS).

### Demarcation of AC-ASPECTS regions

**Figure 1:**
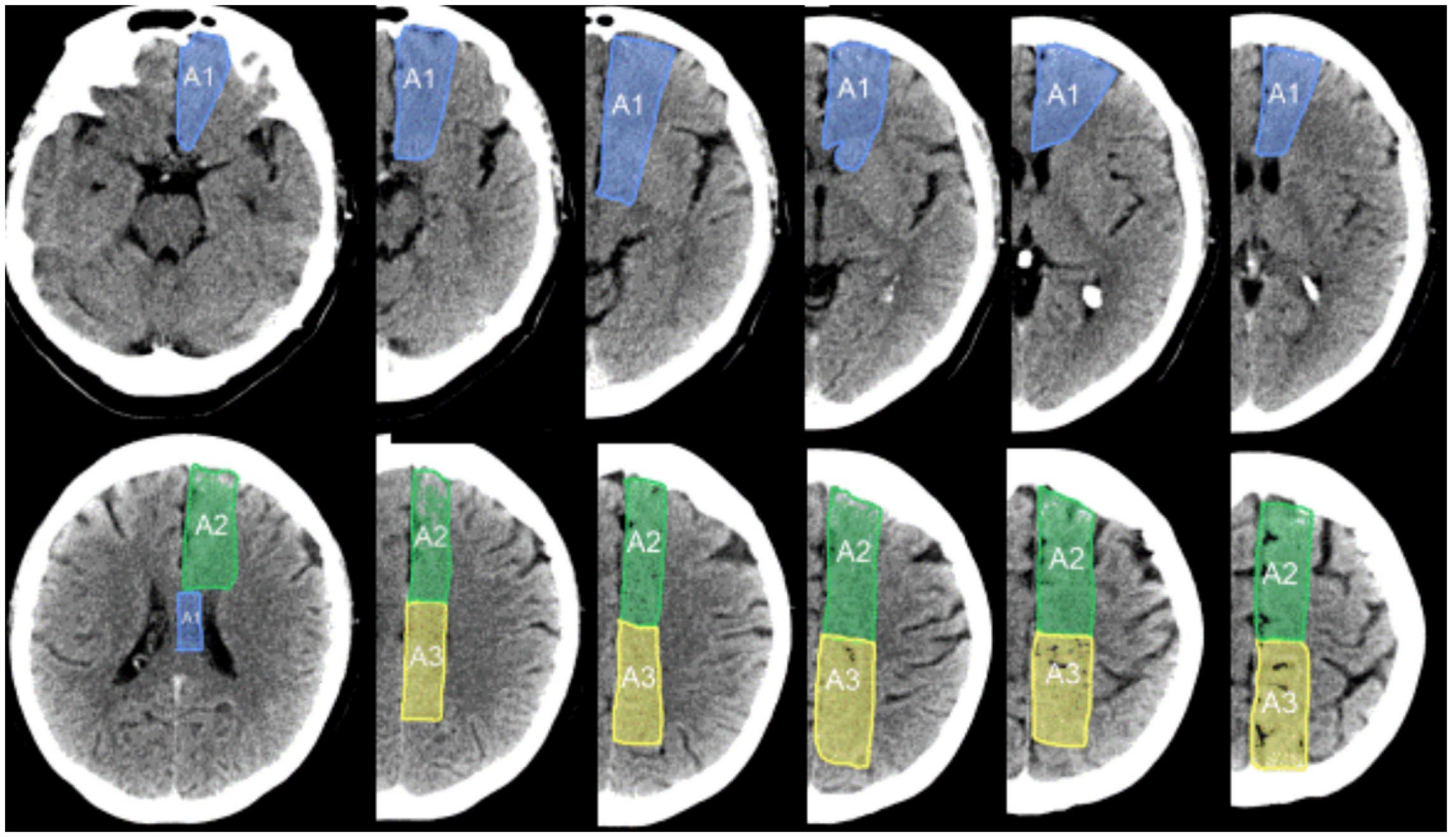
AC-ASPECTS subregions within the anterior cerebral artery territory. The A1 region is the inferior portion of the ACA territory, extending from the lowest slice through the ACA territory up to the highest slice that includes the lateral ventricles. On the highest cut, the A1 region lies only between the ventricles. The A2 region is the anterosuperior portion of the ACA territory. Along the vertical axis, it starts at the highest cut through the lateral ventricles and continues to the highest cut through the top of the cerebrum. Along the anteroposterior axis, the A2 region is the anterior half of the ACA territory on each slice. The A3 region is the posterosuperior portion of the ACA territory. Like the A2, along the vertical axis, it starts at the highest cut through the lateral ventricles and continues to the highest cut through the top of the cerebrum. Along the anteroposterior axis, the A3 region is the posterior half of the ACA territory on each slice.

The volume of the ACA territory is approximately one-third of the volume of the MCA territory (18). The MC-ASPECTS divides the MCA territory into 10 component regions. In order to give proportionate weighting to ACA fields as to MCA fields, the AC-ASPECTS divides the ACA territory into three component regions: A1, A2, and A3. As shown in Figure 1, on standard axial computed tomography slices, the A1 region delineates the inferior portion of the ACA territory, extending from the lowest CT slice up to the highest slice that includes the lateral ventricles. On the highest cut, it lies only between the ventricles. The A2 region is the anterosuperior portion of the ACA territory. Along the vertical axis, it starts at the highest cut through the lateral ventricles and continues to the highest cut through the top of the cerebrum. Along the anteroposterior axis, the A2 region is the anterior half of the ACA territory on each slice. The A3 region is the posterosuperior portion of the ACA territory. Like the A2, along the vertical axis, it starts at the highest cut through the lateral ventricles and continues to the highest cut through the top of the cerebrum. If all ACA regions are normal, the ACA-ASPECTS score is 3. One point is subtracted from 3 for each abnormal region.

### Demarcation of the ACh-ASPECTS region

Of the regions supplied by the ACh, several fall within existing ASPECTS topographies (22). The MC-ASPECTS regions include the posterior limb of the internal capsule and the medial area of the pallidum. The PC-ASPECTS regions include the lateral geniculate body. The ACh-ASPECTS assigns 1 point to spare the remaining, otherwise unassessed region - the mesial temporal lobe (amygdala-hippocampal field), as shown in Supplementary Figure S1.

### Total H-ASPECTS score

The total H-ASPECTS score of a cerebral hemisphere is a 16-point scale that combines the four territorial-specific ASPECTS scores, including the MC-ASPECTS (10 regions), the supratentorial segments of the PC-ASPECTS (two regions: thalamus (THAL) and occipital), the AC-ASPECTS (three regions), and the ACh-ASPECTS (one region). One point is subtracted from 16 for each abnormal region.

### Prospective validation study

The prospective validation cohort study of the ACA-ASPECTS, ACh-ASPECTS, and H-ASPECTS scores was performed in a sample of 50 consecutive patients with confirmed acute ischemic stroke (IS) admitted to Ronald Reagan - University of California, Los Angeles Medical Center. Inclusion criteria for the study were: (1) diagnosis of acute IS on CT or MRI, (2) presentation within 24 h of last known well, (3) occlusion of the cervical or intracranial ICA, M1-MCA, or ACA on initial CTA or MRA. The exclusion criterion was transferred to another acute care hospital, precluding clinical outcome assessment.

For each patient, data were abstracted regarding demographics (age, sex), medical history (e.g., hypertension and diabetes), initial systolic and diastolic blood pressure in the ED, initial National Institutes of Health Stroke Scale (NIHSS) score, the pre-stroke modified Rankin Scale (mRS) score, time from last known well to first parenchymal imaging, time from first imaging to 24 h (−12 h to +24 h) follow-up imaging, reperfusion therapy with intravenous thrombolysis alone, endovascular thrombectomy alone, or both; and hemicraniectomy. If patients had more than one follow-up imaging study performed between 12 and 48 h after initial imaging, the scan closest to 24 h was analyzed. For patients undergoing EVT, the expanded thrombolysis in cerebral infarction (eTICI) score at the procedure end was recorded. Clinical outcomes analyzed were: global disability on the mRS at discharge; discharge destination (home, acute rehabilitation facility, skilled nursing facility, long-term acute care, hospice, or inpatient mortality); and ambulatory status at discharge (unassisted, with assistance, non-ambulatory, or death).

### Imaging

Standard non-helical non-contrast CT (NCCT) was performed on a multislice CT scanner (GE Medical Systems or Siemens) using 120 kV, 170 mAs and 5-mm slice thickness. Coverage was from skull base to vertex, with continuous axial slices parallel to the orbitomeatal line. Standard diffusion-weighted MRI (DWI), as well as ADC, FLAIR, and GRE sequences, were obtained on 1.5-T or 3-T Siemens MR systems equipped with echo planar imaging data acquisition capability designed to obtain rapid diffusion images. Diffusion imaging was performed using a slice thickness of 5 mm with no interslice gap and two levels of diffusion sensitization (b = 0, 1,000 s/mm^2^).

### Imaging analyses

On CT, one point each was subtracted for early ischemic changes (parenchymal hypoattenuation or focal swelling) in each of the defined regions. On MRI, one point each was subtracted for early ischemic changes (hyperintensity on DWI) in ≥20% of each of the defined regions. The images were assessed with knowledge of the side affected but without knowledge of the baseline stroke severity, site of occlusion, or clinical outcome. Baseline scans were assessed without knowledge of 24 h scans; 24 h scans were assessed with knowledge of baseline scans. Each scan was independently scored for AC-ASPECTS, ACh-ASPECTS, PC-ASPECTS, MC-ASPECTS, and H-ASPECTS by three levels of practitioner: one general neurology resident (MPR), one stroke fellow (ST), and one senior stroke neurologist (JLS). Differences were resolved by consensus discussion.

### Statistical analyses

Numerical data were descriptively presented as mean ± standard deviation for parametric data and median with interquartile range (25th percentile–75th percentile) for non-parametric data. Categorical variables were described as the number and percentage of patients. Inter-rater agreement on the AC-ASPECTS, ACh-ASPECTS, and H-ASPECTS was assessed separately for arrival scans and the 24-h scans using joint probability of agreement and the kappa statistic. Kappa values ≤0 were considered to indicate no agreement, 0.01–0.20 as none to slight, 0.21–0.40 as fair, 0.41–0.60 as moderate, 0.61–0.80 as substantial, and 0.81–1.00 as almost perfect agreement (23). Correlation coefficients were calculated for the association of the ASPECTS scales with clinical outcomes. In sample size calculations for a projected correlation of 0.6, the sample size of 50 patients provided 95% power to narrow the two-way 95% confidence interval around the point estimate to 0.15.

## Results

The characteristics of the 50 consecutive IS patients meeting study entry criteria are shown in Table 1. No patients were excluded due to transfer to another acute hospital or missing data. The age was 74.8 years (±14.0), 60% were female, and the baseline NIHSS was 15.5 (IQR 9–20). Occlusion locations were the ICA at 34% (cervical ICA at 16%, intracranial ICA alone at 2%, and carotid T at 16%), M1-MCA at 58%, and ACA at 8%. The time from the last known well to the first imaging was a median of 393 min (IQR 114–851) and from the last known well to follow-up imaging, 32 h (IQR 29–41). A total of 80% of patients received reperfusion therapy, including EVT alone in 48%, IVT alone in 8%, and both in 24%. Hemicraniectomy was performed in 4% of patients.

**Table 1:** Patient characteristics.

Age, y - mean (±SD)	74.8 (±14.0)
Sex, Female - *N* (%)	30 (60)
NIHSS	
Mean (±SD)	14.8 (±7.5)
Median (IQR)	15.5 (9–20)
ED BP - mean (±SD)	
Systolic	147.7 (±29.1)
Diastolic	83.6 (±17.8)
Medical history - *N* (%)	
Hypertension	33 (66)
Diabetes	15 (30)
Dyslipidemia	24 (48)
Atrial fibrillation	21 (42)
Coronary artery disease	9 (18)
Prior ischemic stroke	1 (2)
Prior smoking	0 (0)
Pre-stroke mRS	
Mean (±SD)	1.2 (±1.4)
Median (IQR)	1 (0–2)
Time LKW to 1^st^ imaging, median (IQR)	6 (2–15)
Time LKW to 2^nd^ imaging, median (IQR)	32 (29–41)
Type of 1st imaging (*n* = 50)	
CT, *n* (%)	40 (80)
MR, *n* (%)	10 (20)
Type of 2nd imaging (*n* = 46)	
CT, *n* (%)	11 (24)
MR, *n* (%)	35 (76)
Site of occlusion - *N* (%)	
Cervical ICA	8 (16)
Intracranial ICA	1 (2)
Carotid T	8 (16)
M1-MCA	29 (58)
ACA	4 (8)
Treatment - *N* (%)	
None	9 (18)
IVT alone	4 (8)
EVT alone	24 (48)
IVT + EVT	12 (24)
Hemicraniectomy	2 (4)
eTICI - N (%) (EVT *N* = 36)	
0	1 (3)
1	1 (3)
2A	2 (6)
2B	10 (28)
2C	7 (19)
3	11 (31)
Discharge destination - *N* (%)	
Home	11 (22)
Acute rehabilitation facility	19 (38)
Skilled nursing facility	7 (14)
Long-term acute care	2 (4)
Hospice	4 (8)
In-hospital mortality	7 (14)
Ambulatory status at discharge - *N* (%)	
Ambulatory unassisted	13 (26)
Ambulatory with assistance	20 (40)
Non-ambulatory	10 (20)
In-hospital mortality	7 (14)
mRS at discharge - *N* (%)	
0	3 (6)
1	2 (4)
2	1 (2)
3	7 (14)
4	20 (40)
5	10 (20)
6	7 (14)

**Figure 2:**
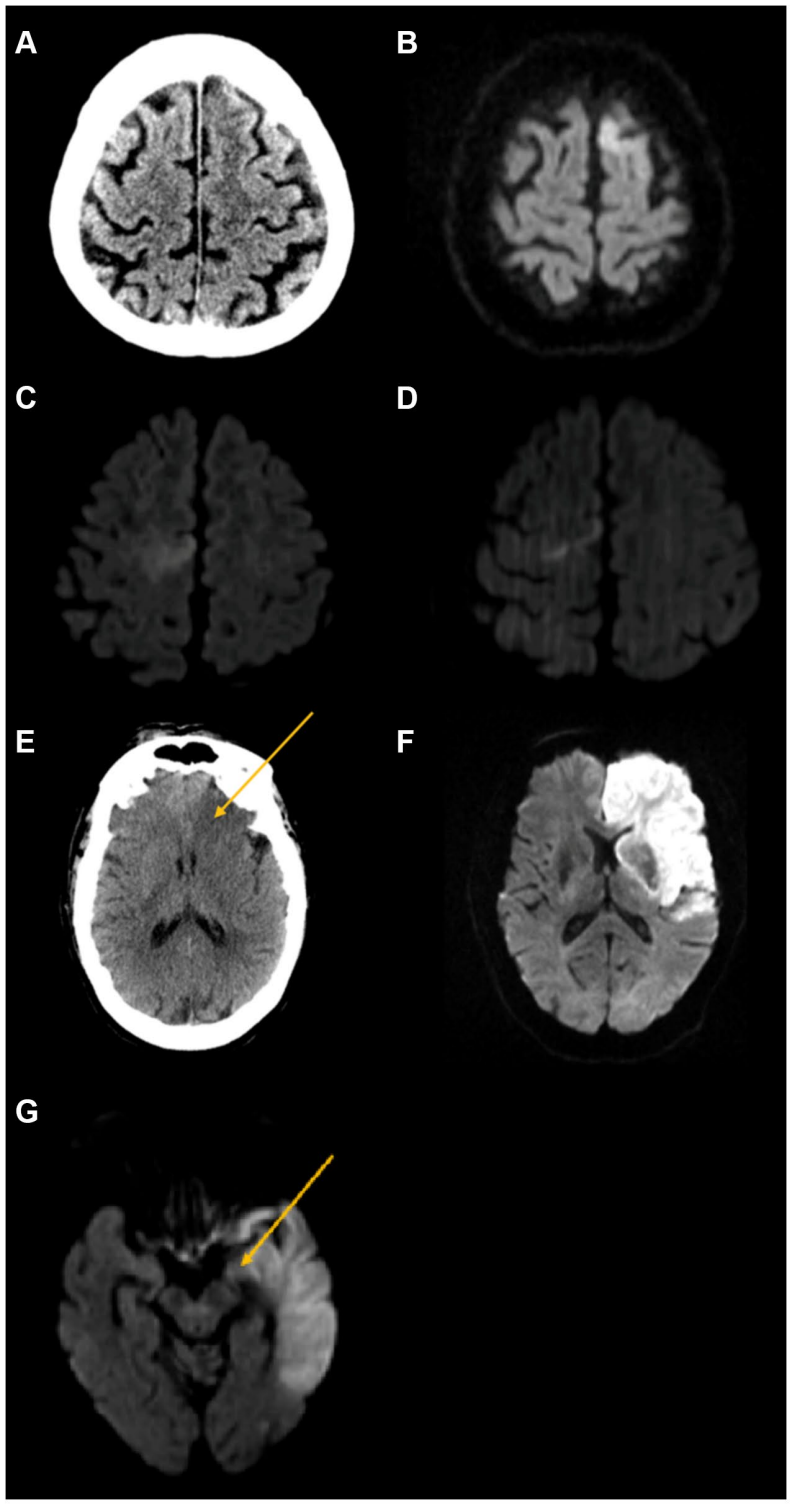
Illustrative cases. First row: Isolated A2 ACA region abnormality. A 64-year-old man presented with right leg sensory changes and severe aphasia, initial NIHSS 3, and CTA showing left ACA occlusion. He was treated with IV alteplase. **(A)** Initial non-contrast CT shows no area of hypodensity (AC-ASPECTS 3 and MC-ASPECTS 10). **(B)** Follow-up DWI MRI shows an abnormality in the left A2 ACA region (AC-ASPECTS 2 and MC-ASPECTS 10). Second row: Isolated A3 ACA region abnormality. **(C)** A 60-year-old woman presented with sudden onset left lower extremity weakness, initial NIHSS 3, and MRA showing right ACA occlusion. She was treated with IV alteplase. Initial DWI MRI shows right A3 ACA region abnormality region (AC-ASPECTS 2 and MC-ASPECTS 10). **(D)** Follow-up DWI MRI also shows right A3 ACA region abnormality (AC-ASPECTS 2 and MC-ASPECTS 10). Third row: Extensive hemispheric abnormality. An 88-year-old man presented with altered mental status, right-sided weakness, left-sided forced gaze deviation, aphasia, and dysarthria, initial NIHSS 18, and CTA showing left ICA occlusion. He was treated with supportive care. **(E)** Initial non-contrast CT shows abnormal hypodensity in the left A1 region (arrow), as well as three MC-ASPECTS regions (H-ASPECTS 12 and C-ASPECT 7). **(F)** Follow-up DWI MRI shows an abnormality in all three AC-ASPECTS regions (A1, A2, and A3) as well as eight MC-ASPECT regions (H-ASPECTS 5 and MC-ASPECTS 2). Fourth row: AChA region abnormality. An 81-year-old woman was transferred from another hospital after intubation for aphasia and right-sided weakness, initial NIHSS 21, and MRA shows left internal carotid artery T occlusion. She was treated with supportive care. **(G)** Initial DWI MRI shows left AChA region abnormality (arrow), as well as 10 MC-ASPECTS regions and 1 PC-ASPECTS region (thalamus) (H-ASPECTS 4 and MC-ASPECTS 0). Follow-up imaging was not obtained.

Concurrence rates among scan interpreters are shown in Table 2 and illustrative cases are shown in Figure 2. Concurrence rates were higher between the two most experienced raters (Attending-Fellow) but also were considerable in dyadic comparisons with the least experienced rater (resident). For the three individual ACA subregions (A1, A2, and A3), the Attending-Fellow raters showed exact agreement in 94–96% of patients and kappa rates of 0.48–0.54 (moderate) on initial images and exact agreement in 89–100% and kappa rates of 0.55 to 1.0 (moderate to almost perfect) on follow-up images. For the total territory AC-ASPECTS score, the Attending-Fellow raters showed exact agreement in 94% of patients and weighted kappa rates of 0.74 (substantial) on the initial images and exact agreement in 97% and weighted kappa 0.62 (substantial) on follow-up images. The attending-fellow rater exact agreement rates for total AC-ASPECTS scores exceeded those for total MC-ASPECTS scores; the weighted kappa values were comparable for the total AC-ASPECTS and total MC-ASPECTS scores. For the total hemispheric H-ASPECTS score, the Attending-Fellow raters showed exact agreement in 67% of patients and weighted kappa rates of 0.73 (substantial) on initial images and showed exact agreement in 74% of patients and weighted kappa rates of 0.73 (substantial) on follow-up images. Attending-Fellow rater agreement rates and kappa values were comparable for the total H-ASPECTS scores and MC-ASPECTS scores. Inter-rater concurrence rates were similar for CT ratings and for MR ratings, although analysis power was limited by low numbers of initial MR scans and follow-up CT scans (Supplementary Tables S1A,B).

**Table 2:** Concurrence rates.

Raters	Measure of concurrence	A1 region	A2 region	A3 region	Total AC-ASPECTS score	Total ACh-ASPECTS score	Total MC-ASPECTS score	Total PC-ASPECTS score	Total H-ASPECTS
Initial imaging									
Attending vs. fellow	Agreement rate	94	96	96	94	100	72	100	67
	Kappa	0.54	0.48	0.48	0.74	1	0.65	1	0.73
Attending vs. resident	Agreement rate	84	88	96	80	100	58	90	55
	Kappa	0.25	0.20	0.48	0.41	1	0.49	0.00	0.61
Fellow vs. resident	Agreement rate	90	92	96	86	100	50	90	50
	Kappa	0.5	0.47	0.48	0.57	1	0.40	0.00	0.50
Follow-up imaging									
Attending vs. Fellow	Agreement rate	91	100	98	87	100	39	93	74
	Kappa	0.55	1	0.90	0.62	1	0.71	0.38	0.73
Attending vs. Resident	Agreement rate	98	93	91	83	100	53	91	74
	Kappa	0.87	0.76	0.55	0.53	1	0.46	0.30	0.73
Fellow vs. Resident	Agreement rate	93	93	93	91	100	33	91	33
	Kappa	0.63	0.76	0.63	0.75	1	0.23	0.30	0.49

### Entire cohort

After consensus discussion, across the entire cohort, AC-ASPECTS abnormality was deemed present in seven (14%) patients (including six patients on initial imaging and seven on follow-up imaging), and ACh-ASPECTS abnormality was present in one (2%) patient (present on both initial and follow-up imaging). Considering AC-ASPECTS subregions, on initial imaging, 6% of patients had A1, 4% of patients had A2, and 4% of patients had A3 involvement. On follow-up imaging, 9% of patients had A1, 13% of patients had A2, and 9% of patients had A3 involvement. The total AC-ASPECTS score on initial imaging was median 3 (IQR 3–3) and mean 2.9 (± 0.4). The total AC-ASPECTS score on follow-up imaging was median 3 (IQR 3–3) and mean 2.7 (± 0.8). AC-ASPECTS abnormalities occurred in isolation, without accompanying MC-ASPECTS abnormalities, in 4% of patients and concurrently with MC-ASPECTS abnormalities in 8%.

Over the entire cohort, on initial imaging, the H-ASPECTS score was a median of 15 (IQR 12–16) and a mean of 13.7 (± 2.6), while the MC-ASPECTS score was 9 (IQR 6–10) and a mean of 7.9 (± 2.5). On follow-up imaging, the H-ASPECTS score was a median of 13 (IQR 10.25–14) with a mean of 12.0 (± 3.2), while the MC-ASPECTS score was 7 (IQR 4–9) with a mean of 6.3 (± 2.9). Among all patients, abnormalities on the H-ASPECTS that were not also present on the MC-ASPECTS occurred on the initial scan in 10% and on the follow-up scan in 15%. Considering patients with ACA or ICA occlusions, abnormalities on the H-ASPECTS not also present on the MC-ASPECTS occurred on the initial scan in 24% and on the follow-up scan in 37%. The frequency of involvement of individual H-ASPECTS regions on baseline and follow-up scans is shown in Supplementary Table S2. Supplementary Table S3 shows the mean/median AC-ASPECTS, MC-ASPECTS, PC-ASPECTS, ACH-ASPECTS, and H-ASPECTS for all patients.

### Occlusion site subgroups

Involvement frequency of ASPECTS regions among patients subgrouped by vascular occlusion sites is shown in Table 3. Involvement of regions A1, A2, and A3 occurred only with ACA and ICA occlusions and not MCA M1 occlusions. Involvement of ACh and THAL occurred only with ICA occlusions and not MCA M1 or ACA occlusions. For the different occlusion sites, H-ASPECTS were: ICA 12 (11–14); M1-MCA 15 (13–16); ACA 15.5 (15–16); MC-ASPECTS scores were: ICA 7 (5–8); M1-MCA 9 (7–10); and ACA 10 (10–10).

**Table 3:** ASPECTS regions involvement by site of occlusion.

ASPECTS scale		Region	Site of occlusion					
			ACA		MCA M1		ICA	
			First scan *N* = 4 (%)	Second scan *N* = 4 (%)	First scan *N* = 29 (%)	Second scan *N* = 26 (%)	First scan *N* = 17 (%)	Second scan *N* = 15 (%)
H-ASPECTS	AC-ASPECTS	A1	1 (25)	2 (50)	0	0	2 (11.8)	2 (13.3)
		A2	0	3 (75)	0	0	2 (11.8)	2 (13.3)
		A3	1 (25)	2 (50)	0	0	1 (5.9)	3 (20)
	ACh-ASPECTS	ACh	0	0	0	0	1 (5.9)	0
	MC-ASPECTS	C	0	0	8 (27.5)	15 (57.7)	10 (58.8)	11 (73.3)
		IC	0	0	3 (10.3)	4 (15.4)	6 (35.3)	6 (40)
		L	0	0	8 (27.5)	17 (65.4)	12 (70.6)	11 (73.3)
		Ins	0	0	12 (41.3)	16 (61.5)	10 (58.8)	10 (66.7)
		M1	0	0	2 (6.9)	4 (15.4)	5 (29.4)	8 (53.3)
		M2	0	0	5 (17.2)	11 (42.3)	4 (23.5)	8 (53.3)
		M3	0	0	2 (6.9)	2 (7.7)	1 (5.9)	3 (20)
		M4	0	0	2 (6.9)	4 (15.4)	4 (23.5)	7 (46.7)
		M5	0	0	5 (17.2)	12 (46.2)	6 (35.3)	9 (60)
		M6	0	0	0	4 (15.4)	1 (5.9)	3 (20)
	PC- ASPECTS	Thalamus	0	0	0	0	1 (5.9)	1 (6.7)
		Occipital	0	0	0	1 (3.8)	0	0

With regard to clinical outcomes, among patients with ACA and ICA occlusions, H-ASPECTS scores were more strongly associated than MC-ASPECTS scores with disability level at discharge, ambulatory status at discharge, discharge destination, and combined inpatient mortality and discharge to hospice in dichotomized (Table 4A) and ordinal (Table 4B and Supplementary Figure S2) analyses. In contrast, among patients with MCA occlusions, H-ASPECTS scores and MC-ASPECTS scores were equally strongly associated with these clinical outcomes (Supplementary Table S4). Considering the area under the curve for dichotomized outcomes, on initial scans, H-ASPECTS scores were again more strongly associated than MC-ASPECTS scores with all four clinical outcomes; on follow-up scans, H-ASPECTS scores were nominally more strongly associated with combined inpatient mortality and discharge to hospice and MC-ASPECTS scores were nominally more strongly associated with disability level at discharge, ambulatory status at discharge, and discharge destination (Supplementary Table S5). Among patients with ACA occlusions, AC-ASPECTS was more strongly associated than MC-ASPECTS with these outcomes.

**Table 4A:** H-ASPECTS, MC-ASPECTS, and clinical outcomes in patients with ICA or ACA occlusions.

Outcome	Imagine score	First scan			Second scan		
		Outcome = Yes	Outcome = No	*p*-value	Outcome = Yes	Outcome = No	*p*-value
mRS 0–3 at discharge	H-ASPECTS	14.7 (2.5)	12.3 (3.0)	0.1	13.7 (2.6)	9.8 (4.2)	0.09
		16	12	0.1	14.5	10	0.08
	MC-ASPECTS	8.7 (2.5)	6.8 (2.8)	0.2	8.2 (2.9)	4.7 (3.6)	0.8
		10	7	0.2	9.5	4	0.1
Discharge home	H-ASPECTS	15.7 (0.5)	12.1 (2.9)	0.02	15 (0.8)	9.5 (3.9)	0.01
		16	12	0.01	15	10	0.01
	MC-ASPECTS	10 (0)	6.5 (2.7)	0.02	9.7 (0.5)	4.3 (3.3)	0.005
		10	7	0.01	10	4	0.01
Discharge home or acute rehab	H-ASPECTS	13.6 (2.1)	11.4 (3.9)	0.1	12.3 (2.7)	7.0 (4.7)	0.006
		14	12	0.2	13	5	0.02
	MC-ASPECTS	7.85 (2.1)	6.1 (3.4)	0.1	6.8 (2.9)	2.7 (3.7)	0.01
		8	6.5	0.2	7	2	0.02
Discharge ambulatory unassisted	H-ASPECTS	14.7 (2.5)	12.3 (3.0)	0.1	13.7 (2.6)	9.8 (4.2)	0.09
		16	12	0.1	14.5	10	0.08
	MC-ASPECTS	8.7 (2.5)	6.8 (2.8)	0.2	8.2 (2.9)	4.7 (3.6)	0.08
		10	7	0.2	9.5	4	0.1
Discharge ambulatory unassisted or assisted	H-ASPECTS	13.7 (2.2)	11.2 (3.7)	0.07	12.5 (2.9)	6.7 (3.9)	0.002
		14	12	0.1	13	5	0.009
	MC-ASPECTS	7.8 (2.1)	6.1 (3.4)	0.1	6.8 (2.9)	2.7 (3.7)	0.01
		8	6.5	0.2	7	2	0.02
Death or discharge to hospice	H-ASPECTS	10 (3.5)	13.6 (2.4)	0.01	6.0 (1.73)	11.5 (3.9)	0.03
		12	14.5	0.04	5	12.5	0.04
	MC-ASPECTS	5.2 (3.1)	7.8 (2.4)	0.06	2.0 (0)	6.1 (3.6)	0.07
		6	8.5	0.1	2	6.5	0.07

**Table 4B:** Correlation between MC-ASPECTS, H-ASPECTS and clinical outcomes in patients with ICA and ACA occlusions.

Scan timing	Rating scale	mRS level (full) (0,1,2,3,4,5,6)	mRS level (severe-weighted) (0–2,3,4,5,6)	Ambulatory status*	Discharge destination**
Scan 1	MC-ASPECTS	−0.29	−0.27	−0.30	−0.28
	H-ASPECTS	−0.38	−0.36	−0.4	−0.31
Scan 2	MC-ASPECTS	−0.50	−0.52	−0.58	−0.42
	H-ASPECTS	−0.55	−0.60	−0.67	−0.44

^*^Ambulatory status: 4-level ordinal variable: ambulate independently/ambulate with assistance/non-ambulatory/dead. ^**^Discharge destination: 4-level ordinal variable: home/acute rehab/SNF/dead or hospice.

## Discussion

This study developed and validated three new IS brain parenchymal imaging rating scales, AC-ASPECTS, ACh-ASPECTS, and H-ASPECTS, that extend semi-quantitative ischemic injury assessment to regions unaddressed by the existing MC-ASPECTS and PC-ASPECTS instruments. These new atlas-based scores allow the characterization of injury extent for infarctions confined to the ACA territory, infarctions confined to the ACh territory amygdala-hippocampal field, and cumulative injury degree throughout the entire human cerebral hemisphere. Inter-rater agreement on both presenting and follow-up images was high for all three new scales. Among patients with occlusions of the ICA, H-ASPECTS scores were more strongly associated than MC-ASPECTS scores with the level of disability at discharge, ambulatory status at discharge, discharge destination, and combined inpatient mortality and discharge to hospice. Among patients with occlusions of the ACA, both the AC-ASPECTS and H-ASPECTS correlated more strongly than the MC-ASPECTS with clinical outcomes.

The AC-ASPECTS, ACh-ASPECTS, and H-ASPECTS substantially increase the amount of information provided by the ASPECTS rating strategy in patients with acute IS due to ACA, ACh, and ICA occlusions, which collectively account for more than one in five ISs due to large and medium vessel occlusions in the anterior circulation (24, 25). Isolated ACA infarctions account for 1–3% of ISs in large cohort studies (26–28) and can produce substantial disability, with common clinical manifestations of lower extremity predominant hemiparesis, urinary incontinence, apathy, and aphasia (26–28). Being able to characterize ACA infarct extent with a pragmatic, semi-quantitative ASPECTS scale provides a pragmatic method to rate ACA core extent rapidly prior to intravenous thrombolysis in current clinical practice and prior to endovascular thrombectomy in randomized trials of catheter intervention for distal, medium vessel occlusions.

ICA occlusions, which account for 6–15% of ISs (29), may now be more accurately characterized by the use of the H-ASPECTS scale, which captures not only the 10 MC-ASPECTS regions that may be involved but the 6 additional AC-ASPECTS, ACh-ASPECTS, and PC-ASPECTS regions also often affected (29–32). As the MCA territory supplies only approximately 54% of the human cerebral hemisphere (20), the MC-ASPECTS scale alone interrogates a markedly incomplete fraction of the entire supratentorial brain volume that can be compromised by ICA occlusions, which can additionally affect the ACA, AChA, and PCA fields. Each of these territories supplies substantial portions of the cerebral hemisphere: ACA approximately 18%, posterior cerebral artery approximately 13%, and ACh approximately 4% (with watershed zones accounting for the remaining approximately 11%) (20–22, 33). In patients with ICA occlusions, the H-ASPECTS scale has the potential to better guide decision-making than the MC-ASPECTS scale alone, as it delineates the extent of injury in its entirety rather than just a bit more than one-half of the potential ICA territory.

Recent randomized trials have demonstrated the benefit of endovascular thrombectomy for select patients with large infarct cores, but the core size was most often assessed incompletely via the use of the MC-ASPECTS alone (1). Reassessment for subgroups of patients who do and do not benefit from thrombectomy is desirable with the use of the H-ASPECTS scale. Of note, in the current study, the MC-ASPECTS scale did exhibit worse scores among patients with ICA compared with MCA occlusion, despite not interrogating non-MCA brain regions. The worse scores likely reflected that MCA field ischemic was more severe in ICA than M1-MCA occlusions due to the compromise of collateral supply from non-MCA to MCA territories. As a result, the MC-ASPECTS scores did indicate a greater injury degree among ICA patients than MCA occlusion patients. Nonetheless, adding scores reflecting injury or sparing of non-MCA fields would, on physiologic grounds, be expected to further improve scale performance in predicting prognosis and responsiveness to thrombectomy. Moreover, the H-ASPECTS scale has the potential to improve the identification of patients who may benefit from hemicraniectomy, as ACA region infarction accompanying MCA infarction in patients with ICA occlusion is an established risk factor for the development of malignant cerebral infarction and the need for surgical intervention (12–14).

This study has limitations. First, the investigation was performed retrospectively at a single academic medical center in a cohort of moderate size. Larger, prospective, multicenter, confirmatory studies are desirable. Second, the current study analyzed patient functional outcomes and vital status at discharge. Long-term outcome studies, especially through 3 months, are needed. Third, while the current study assessed inter-rater reliability among three raters with a wide range of scan assessment experience, performance characterization by more raters, including community practitioners, is recommended. In addition, in consensus discussions to resolve rating differences, the more senior rater(s) may have had greater influence than the more junior rater(s), but there were cases in which the final consensus was in favor of the readings by the more junior reviewer(s). Fourth, large ischemic core patients in the current study were generally not treated with endovascular thrombectomy, reflecting extant national guideline recommendations during the study period that preceded the completion of recent large core randomized trials. Furthermore, as in usual practice, the proportion of patients for whom hemicraniectomy was performed was low. Studies in larger and randomized trial populations are advised to confirm that and to characterize by how much the more encompassing H-ASPECTS, may better aid decision-making regarding these treatments than the MC-ASPECTS alone. Fifth, to match the validation approach of the original MC-ASPECTS study, the population in this initial study of the expanded ASPECTS scale constituted consecutive patients with anterior circulation IS. Further studies in larger numbers of patients, specifically with ICA and ACA occlusions, in whom scale performance would most differ, are desirable. Sixth, the current study assessed as scan raters a broad range of neurologic clinicians with three levels of neurologic training. Further inter-rater reliability studies are needed that encompass a wider range of physicians who perform acute stroke care, including more interventional neuroradiologists, endovascular neurosurgeons, interventional neurologists, diagnostic neuroradiologists, and additional non-interventional stroke neurologists.

## Conclusion

AC-ASPECTS, ACh-ASPECTS, and H-ASPECTS expand the scope of AIS imaging scores and increase correlation with presenting deficits and functional outcomes. This additional information may improve decision-making in patients with large ischemic cores, including endovascular thrombectomy and hemicraniectomy.

## Data availability statement

The original contributions presented in the study are included in the article/Supplementary material, further inquiries can be directed to the corresponding author.

## Ethics statement

Ethical review and approval was not required for the study on human participants in accordance with the local legislation and institutional requirements. Written informed consent from the patients/participants or patients/participants' legal guardian/next of kin was not required to participate in this study in accordance with the national legislation and the institutional requirements.

## Author contributions

MR: Writing – original draft, Writing – review & editing. ST: Writing – original draft, Writing – review & editing. JL: Writing – original draft, Writing – review & editing. DL: Writing – review & editing. JS: Writing – original draft, Writing – review & editing.
