# Supplementary material for: AC-ASPECTS, ACh-ASPECTS, and H-ASPECTS: new imaging scales to assess territorial and total cerebral hemispheric ischemic injury
Source: Front Neurol. 2024 Jul 3;15:1397120. doi: 10.3389/fneur.2024.1397120 (PMC11252022; doi:10.3389/fneur.2024.1397120)
Supplement: Supplementary file 1 [file Data_Sheet_1.docx]

Supplemental Material

**eTable 1A Concurrence Rates – CT scans**

| Raters | Measure of Concurrence | A1  Region | A2  Region | A3  Region | Total AC-ASPECTS  Score | Total ACh-ASPECTS  Score | Total MC-ASPECTS  Score | Total PC-ASPECTS  Score | Total H-ASPECTS |
| --- | --- | --- | --- | --- | --- | --- | --- | --- | --- |
| Initial Imaging | | | | | | | | | |
| Attending vs Fellow | Agreement rate | 93 | 95 | 98 | 95 | 98 | 73 | 100 | 69 |
|  | Kappa | 0.36 | 0.47 | 0.65 | 0.77 | 0.0 | 0.64 | Na | 0.60 |
| Attending vs Resident | Agreement rate | 83 | 90 | 98 | 80 | 100 | 57 | 100 | 59 |
|  | Kappa | 0.13 | 0.28 | 0.65 | 0.40 | NA | 0.46 | Na | 0.48 |
| Fellow vs Resident | Agreement rate | 90 | 95 | 100 | 85 | 98 | 50 | 100 | 53 |
|  | Kappa | 0.45 | 0.64 | 1.0 | 0.51 | 0.0 | 0.39 | Na | 0.42 |
| Follow-Up Imaging | | | | | | | | | |
| Attending vs Fellow | Agreement rate | 91 | 100 | 100 | 91 | 100 | 36 | 100 | 27 |
|  | Kappa | 0.0 | 1 | 1 | 0.71 | 1 | 0.28 | Na | 0.19 |
| Attending vs Resident | Agreement rate | 82 | 91 | 91 | 73 | 100 | 100 | 91 | 73 |
|  | Kappa | 0.0 | 0.74 | 0.62 | 0.40 | 1 | 1 | 0.0 | 0.67 |
| Fellow vs Resident | Agreement rate | 91 | 91 | 91 | 82 | 100 | 36 | 91 | 27 |
|  | Kappa | 0.62 | 0.74 | 0.62 | 0.60 | 1 | 0.28 | 0.0 | 0.18 |

**eTable 1B Concurrence Rates – MRI scans**

| Raters | Measure of Concurrence | A1  Region | A2  Region | A3  Region | Total AC-ASPECTS  Score | Total ACh-ASPECTS  Score | Total MC-ASPECTS  Score | Total PC-ASPECTS  Score | Total H-ASPECTS |
| --- | --- | --- | --- | --- | --- | --- | --- | --- | --- |
| Initial Imaging | | | | | | | | | |
| Attending vs Fellow | Agreement rate | 100 | 100 | 90 | 90 | 100 | 70 | 100 | 60 |
|  | Kappa | 1 | Na | 0.0 | 0.61 | 1 | 0.63 | 1 | 0.54 |
| Attending vs Resident | Agreement rate | 90 | 80 | 90 | 80 | 90 | 60 | 90 | 50 |
|  | Kappa | 0.61 | 0.00 | 0.0 | 0.42 | 0.61 | 0.52 | 0.0 | 0.44 |
| Fellow vs Resident | Agreement rate | 90 | 80 | 80 | 90 | 90 | 50 | 90 | 50 |
|  | Kappa | 0.61 | 0.00 | -0.1 | 0.75 | 0.61 | 0.39 | 0.0 | 0.39 |
| Follow-Up Imaging | | | | | | | | | |
| Attending vs Fellow | Agreement rate | 89 | 100 | 97 | 86 | 100 | 40 | 97 | 29 |
|  | Kappa | -0.44 | 1 | 0.87 | 0.58 | 1 | 0.32 | 0.63 | 0.18 |
| Attending vs Resident | Agreement rate | 89 | 94 | 91 | 86 | 100 | 100 | 94 | 80 |
|  | Kappa | -0.48 | 0.76 | 0.53 | 058 | 1 | 1 | 0.47 | 0.76 |
| Fellow vs Resident | Agreement rate | 94 | 94 | 94 | 94 | 100 | 40 | 97 | 37 |
|  | Kappa | 0.63 | 0.76 | 0.63 | 0.81 | 1 | 0.33 | 0.65 | 0.27 |

**eTable 2A. ASPECTS Hemispheric Regions Involvement Overall**

| Abnormality | First Scan N (%) | Second Scan N (%) |
| --- | --- | --- |
| AC-ASPECTS | 6 (12) | 7 (15.2) |
| MC-ASPECTS | 30 (60) | 37 (80.4) |
| PC- ASPECTS | 1 (2) | 3 (6.5) |
| ACh-ASPECTS | 1 (2) | 0 |
| H-ASPECTS | 32 (64) | 40 (87) |
| ACA + MCA | 4 (8) | 5 (11) |
| ACA + PCA | 0 | 0 |

**eTable 2B. ASPECTS Hemispheric Regions Involvement Overall**

|  | Region | First Scan N (%) | Second Scan N (%) |
| --- | --- | --- | --- |
| AC-ASPECTS | A1 | 3 (6) | 4 (8.7) |
|  | A2 | 2 (4) | 6 (13) |
|  | A3 | 2 (4) | 4 (8.7) |
| MC-ASPECTS | C | 18 (36) | 26 (56.5) |
|  | IC | 9 (18) | 11 (23.9) |
|  | L | 20 (40) | 28 (60.9) |
|  | Ins | 22 (44) | 27 (58.7) |
|  | M1 | 7 (14) | 12 (26.1) |
|  | M2 | 9 (18) | 19 (41.3) |
|  | M3 | 3 (6) | 5 (10.9) |
|  | M4 | 6 (12) | 12 (26.1) |
|  | M5 | 11 (22) | 21 (45.7) |
|  | M6 | 1 (2) | 8 (17.4) |
| PC- ASPECTS | Thal | 1 (2) | 1 (2.2) |
|  | Occip | 0 (0) | 2 (4.3) |
| ACh-ASPECTS | AH | 1 (2) | 0 (0) |

**eTable 3. ASPECTS Total Scores Overall**

| Region | First Scan N (%) | Second Scan N (%) |
| --- | --- | --- |
| AC-ASPECTS | | |
| Mean SD | 2.9 (0.4) | 2.7 (0.8) |
| Median IQR | 3 (3-3) | 3 (3-3) |
| ACh-ASPECTS | | |
| Mean SD | 1 (0.1) | 1 (0) |
| Median IQR | 1 (1-1) | 1 (1-1) |
| MC-ASPECTS | | |
| Mean SD | 7.9 (2.5) | 6.3 (2.9) |
| Median IQR | 9 (6.2-10) | 7 (4.2-8.7) |
| PC-ASPECTS | | |
| Mean SD | 2 (0.1) | 1.9 (0.2) |
| Median IQR | 2 (2-2) | 2 (2-2) |
| H-ASPECTS | | |
| Mean SD | 13.7 (2.7) | 12 (3.2) |
| Median IQR | 15 (12-16) | 13 (10.2-14) |

**eTable 4**: H-ASPECTS vs MC-ASPECTS and Outcomes in All Patients

| Outcome | Imagine Score | First Scan | | | Second Scan | | |
| --- | --- | --- | --- | --- | --- | --- | --- |
|  |  | Outcome = Yes | Outcome = No | P value | Outcome = Yes | Outcome = No | P value |
| mRS 0-3 | H-ASPECTS | 15 (1.8) | 13.2 (2.9) | 0.03 | 13.8 (2.3) | 11.2 (3.4) | 0.01 |
|  |  | 16 (16-14.7) | 14 (15-11) | 0.01 | 14 (15.2-13) | 12 (13.5-6) | 0.01 |
|  | MC-ASPECTS | 9 (1.8) | 7.5 (2.6) | 0.05 | 8 (2.3) | 5.7 (2.9) | 0.01 |
|  |  | 10 | 8 | 0.03 | 9 | 6 | 0.01 |
| Discharge home | H-ASPECTS | 15.2 (1.4) | 13.3 (2.8) | 0.03 | 14.3 (1.9) | 11.2 (3.3) | 0.003 |
|  |  | 16 | 14 | 0.02 | 15 | 12 | 0.003 |
|  | MC-ASPECTS | 9.3 (1.4) | 7.5 (2.6) | 0.03 | 8.5 (2.1) | 5.7 (2.8) | 0.006 |
|  |  | 10 | 8 | 0.01 | 10 | 6 | 0.003 |
| Discharge home or  acute rehab | H-ASPECTS | 14.07 (2.08) | 13.1 (3.3) | 0.2 | 12.8 (2.4) | 10.5 (4.1) | 0.03 |
|  |  | 15 | 14.5 | 0.4 | 13 | 12 | 0.07 |
|  | MC-ASPECTS | 8.17 (2.09) | 7.4 (3) | 0.3 | 7.0 (2.5) | 5.2 (3.3) | 0.01 |
|  |  | 9 | 8.5 | 0.4 | 7 | 6 | 0.08 |
| Discharge ambulatory unassisted | H-ASPECTS | 15 (1.8) | 13.2 (2.8) | 0.03 | 13.8 (2.9) | 11.2 (3.4) | 0.01 |
|  |  | 16 | 14 | 0.01 | 14 | 12 | 0.01 |
|  | MC-ASPECTS | 9 (1.8) | 7.5 (2.6) | 0.05 | 8 (2.3) | 5.7 (2.9) | 0.01 |
|  |  | 10 | 8 | 0.03 | 9 | 6 | 0.01 |
| Discharge ambulatory unassisted  or assisted | H-ASPECTS | 14.4 (1.8) | 12.3 (3.4) | 0.006 | 13 (2.3) | 9.3 (3.9) | 0.001 |
|  |  | 15 | 12 | 0.02 | 13 | 9 | 0.006 |
|  | MC-ASPECTS | 8.5 (1.8) | 6.7 (3.2) | 0.01 | 7.2 (2.3) | 4.2 (3.2) | <0.001 |
|  |  | 9 | 8 | 0.06 | 8 | 3 | 0.004 |
| Death or discharge  to hospice | H-ASPECTS | 12.2 (3.5) | 14.1 (2.2) | 0.03 | 9.9 (3.6) | 12.4 (3.1) | 0.06 |
|  |  | 12 | 15 | 0.06 | 10.5 | 13 | 0.06 |
|  | MC-ASPECTS | 6.8 (3.1) | 8.2 (2.2) | 0.08 | 4.6 (2.6) | 6.7 (2.9) | 0.04 |
|  |  | 8 | 9 | 0.09 | 4.5 | 7 | 0.07 |

**eTable 5. Area Under the Curve for Dichotomized Clinical Outcomes for H-ASPECTS and MC-ASPECTS in Patients with ICA or ACA Occlusions**

|  | mRS 0-3 at discharge (vs mRS 4-6) | Discharge to home or acute rehab (vs other) | Ambulatory with or wo assistance at discharge (vs other) | Inpatient mortality or dc to hospice (vs other) |
| --- | --- | --- | --- | --- |
| H-ASPECTS  initial | 0.74 | 0.65 | 0.70 | 0.81 |
| MC-ASPECTS  initial | 0.71 | 0.64 | 0.64 | 0.75 |
| H-ASPECTS  Follow-up | 0.73 | 0.75 | 0.75 | 0.95 |
| MC-ASPECTS  Follow-up | 0.77 | 0.84 | 0.84 | 0.84 |

**eFigure 1**


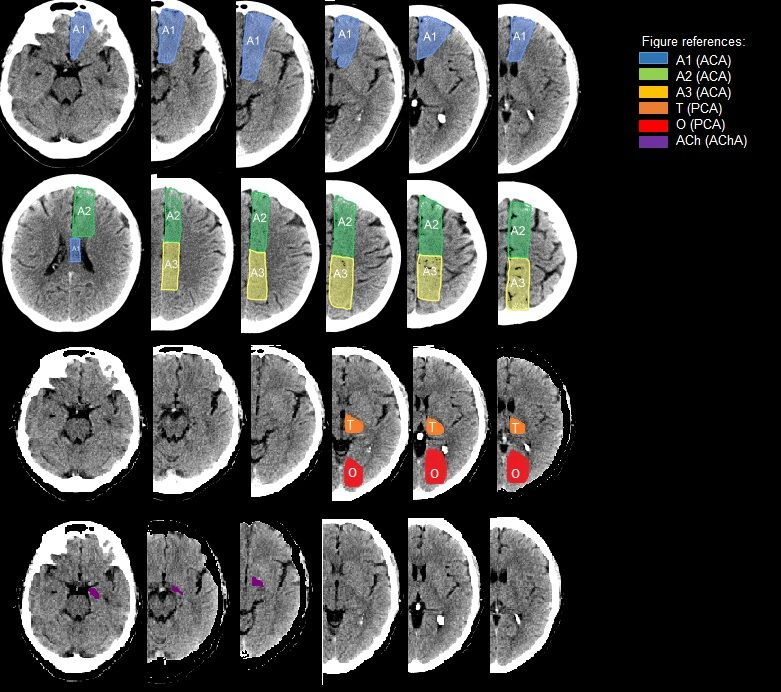


**eFigure 2. Correlation of H-ASPECTS and MC-ASPECTS with Discharge mRS**

| All patient´s correlations | | | |
| --- | --- | --- | --- |
| 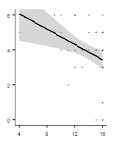 | 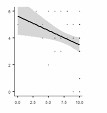 | 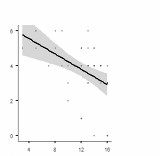 | 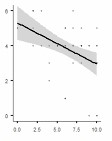 |
| Initial H-ASPECTS | Initial MC-ASPECTS | Second H-ASPECTS | Second MC-ASPECTS |
| ACA+ICA patient's occlusion correlations | | | |
| 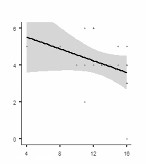 | 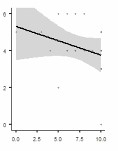 | 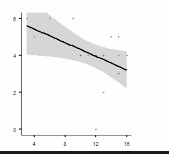 | 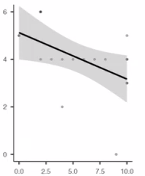 |
| Initial H-ASPECTS | Initial MC-ASPECTS | Second H-ASPECTS | Second MC-ASPECTS |
